# Supplementary material for: Characterization of vaginal lactobacilli from South African women toward the development of a biotherapeutic to optimize the vaginal microbiome
Source: Appl Environ Microbiol. 2026 Aug 21;92(9):e00566-26. doi: 10.1128/aem.00566-26 (PMC13599504; doi:10.1128/aem.00566-26)
Supplement: Supplementary file 1 [file aem.00566-26-s0001.pdf]

Supplementary Figure 1

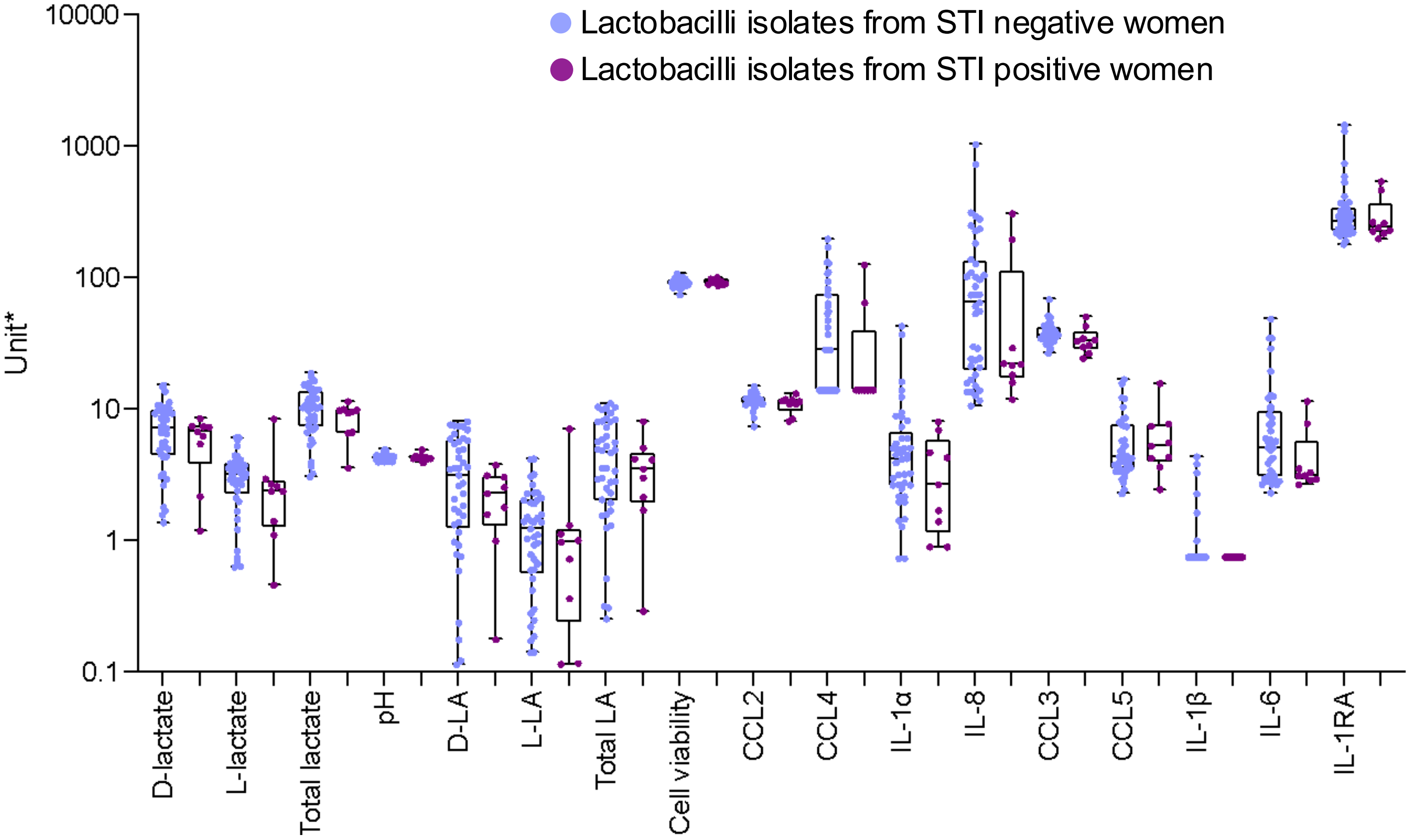

Supplementary Figure 1: Comparison of lactate and lactic acid production, culture acidification, and cytokine responses between lactobacilli isolates from women with or without sexually transmitted infections (STIs). All variables assessed in this study were compared between isolates from STI positive and negative women using Mann Whitney U test. Lactate and pH levels were measured in culture supernatants following 24h incubation of standardised lactobacilli isolates [*Lactobacillus crispatus* (n=29), *Lb. jensenii* (n=8), *Lb. gasseri* (n=7), *Limosilactobacillus vaginalis* (n=3), *Lb. johnsonii* (n=2), *Ligilactobacillus salivarius* (n=1)] and two ATCC reference strains (*Lb. crispatus* ATCC® 33820™ and *Lm. vaginalis* ATCC® 49540™) in de Man, Rogosa and Sharpe broth under anaerobic conditions. For the cytokine assays, these lactobacilli strains were incubated with VK2 cells at 37°C, 5% CO_2 for 24 h. VK2 viability was assessed using trypan blue and culture supernatants were collected for measurement of interleukin (IL)-6, IL-8, IL-1α, IL-1β, IL-1 receptor antagonist (RA), chemokine ligand (CCL)-2 [monocyte chemoattractant protein (MCP)-1], CCL3 [macrophage inflammatory protein (MIP)-1α], CCL4 (MIP-1β) and CCL5 [regulated on activation, normal T cell expressed and secreted (RANTES)] using Luminex. Lactic acid concentrations were calculated using the Henderson-Hasselbalch equation. Boxes represent the interquartile ranges, lines within boxes represent medians and whiskers represent minimum and maximum values. No significant differences were found. *Units are g/L for D-lactate, L-lactate, total lactate, D-lactic acid, L-lactic acid and total lactic acid, pH units for culture acidification, percentage of untreated cells for cell viability and pg/ml for cytokine production.

Supplementary Figure 2

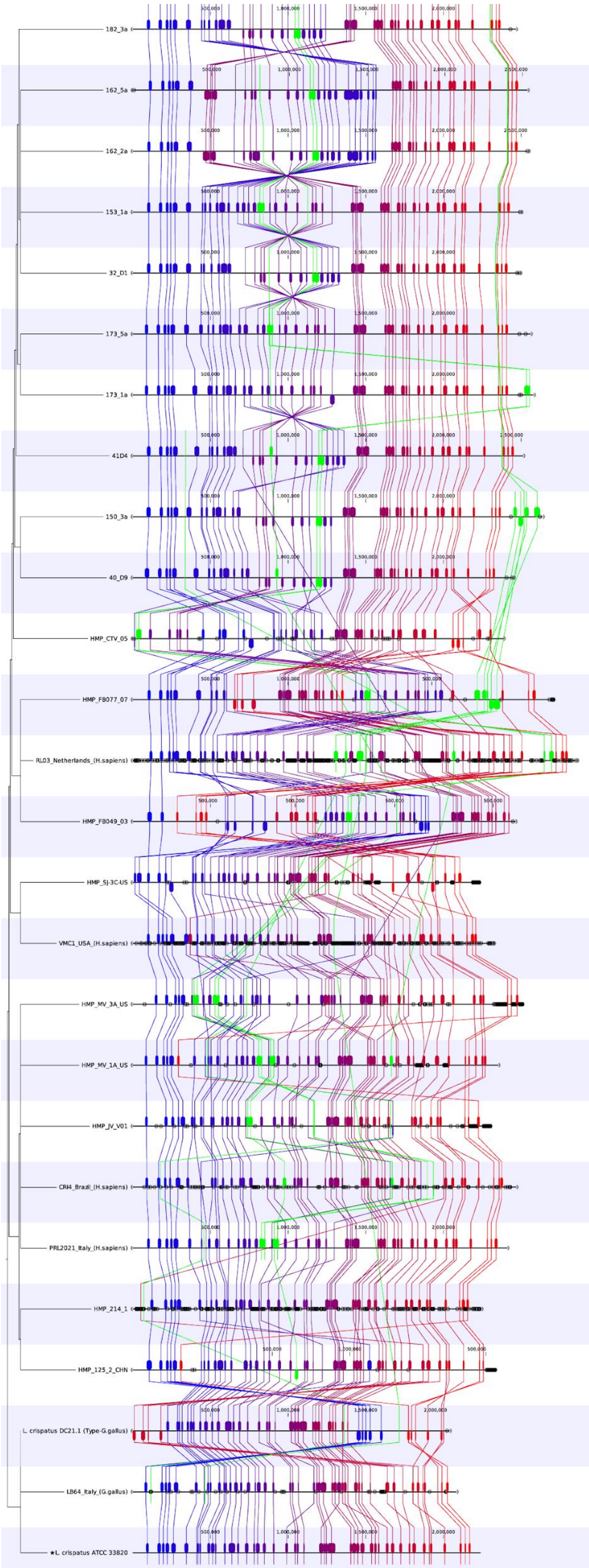

Supplementary Figure 2: Gene block alignment of *Lactobacillus crispatus* genomes. Novel *Lb. crispatus* isolate whole genome sequence data were aligned with the reference *Lb. crispatus* ATCC 33820 strain using the Whole Genome Alignment tool within CLC Genomics. Publicly available *Lb. crispatus* genomes from other geographical regions were included for comparison. Blue, purple and red blocks indicate gene regions possessed by the ATCC 33820 strain. Green blocks are unique to other isolates and not possessed by the ATCC 33820 strain. ATCC: American Type Culture Collection; HMP: Human Microbiome Project; *G. gallus*: *Gallus gallus*; *H. sapiens*: *Homo sapiens*.

Supplementary Figure 3

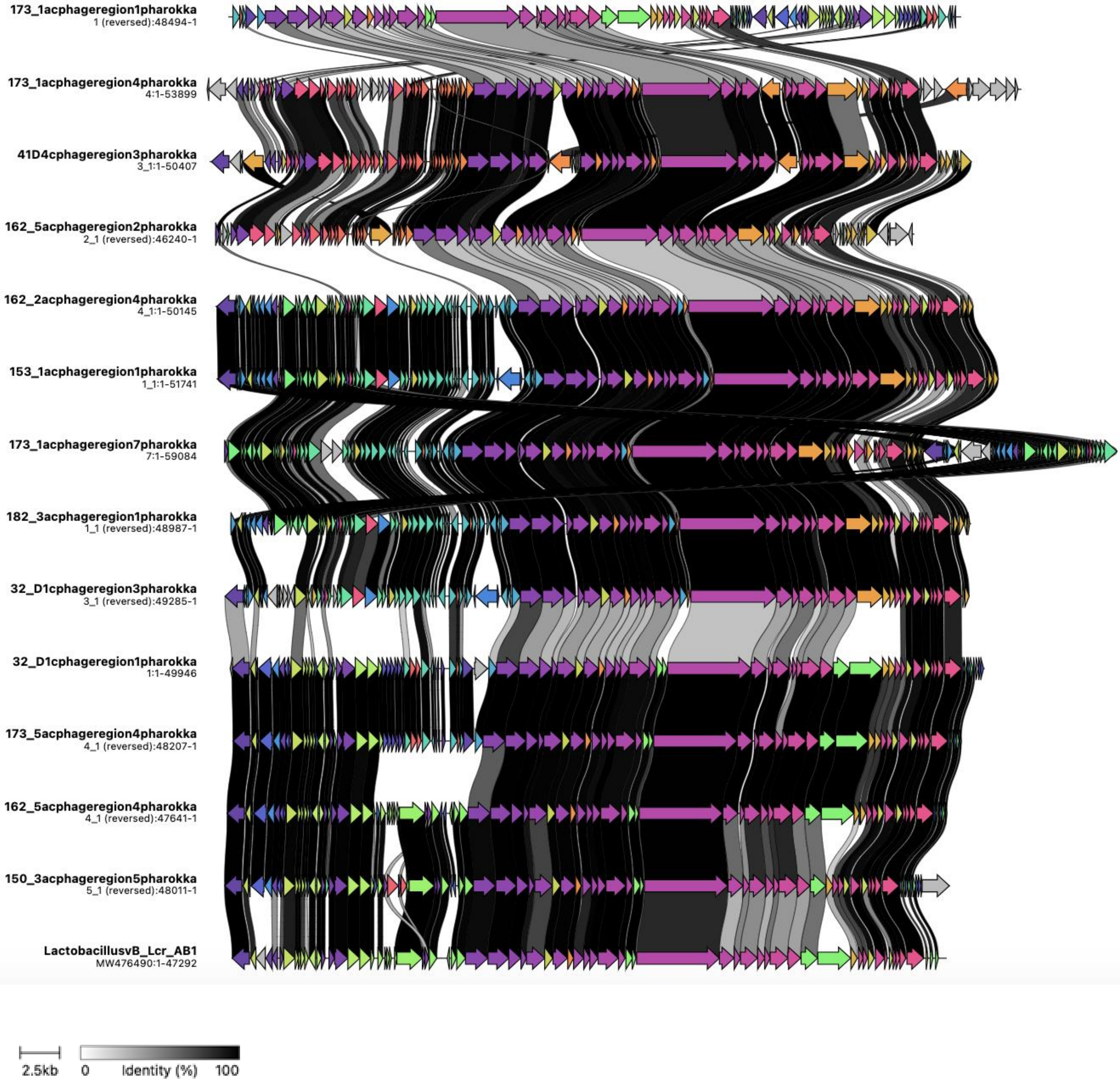

Supplementary Figure 3: Pairwise alignment of gene clusters of selected high-quality *Lactobacillus crispatus* prophage sequences. Selected high-quality *Lb. crispatus* phage sequences obtained from CheckV were aligned with the corresponding top CheckV prophage hit Lactobacillus phage vB_Lcr_AB1 using the gene cluster comparison figure generator clinker. Unique colours indicate a different similarity group. The lines indicate gene similarity and are shaded according to the sequence identity (0% white, 100% black).

Supplementary Figure 4

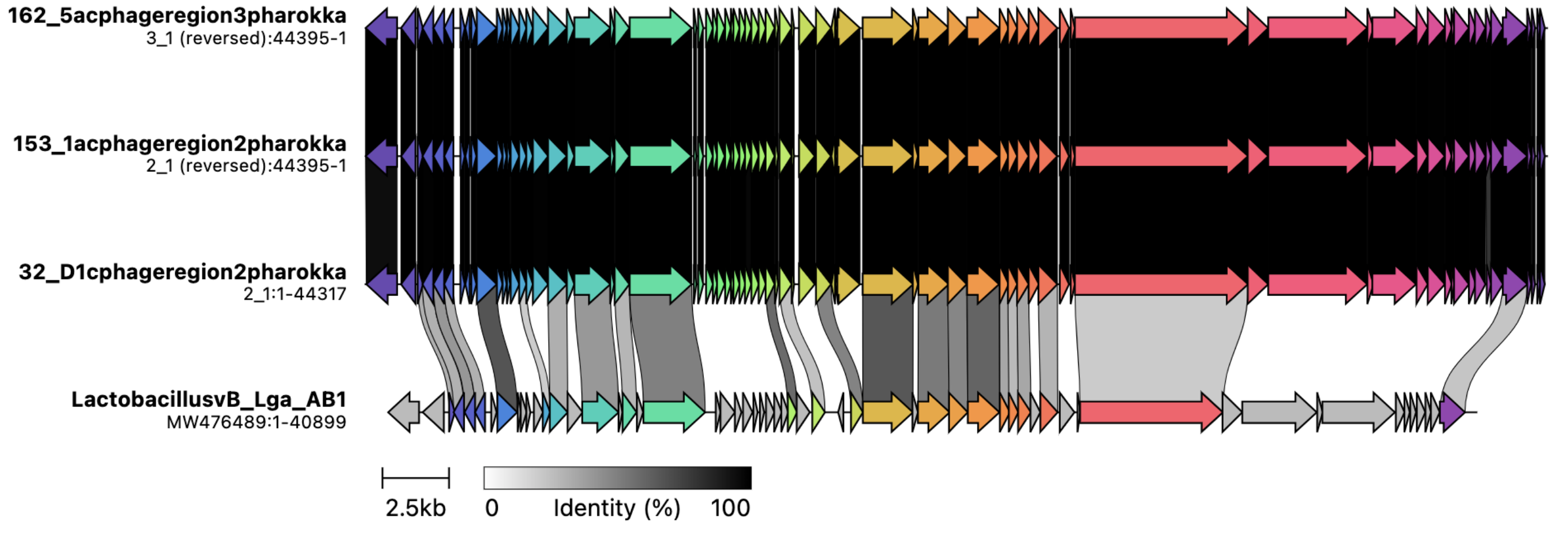

Supplementary Figure 4: Pairwise alignment of gene clusters of high-quality *Lactobacillus crispatus* prophage sequences. Selected high-quality *Lb. crispatus* phage sequences obtained from CheckV were aligned with the corresponding top CheckV prophage hit Lactobacillus phage vb_Lga_AB1 using the gene cluster comparison figure generator clinker. Unique colours indicate a different similarity group. The lines indicate gene similarity and are shaded according to the sequence identity (0% white, 100% black).
